# Infiltrating neutrophils increase bladder cancer cell invasion *via* modulation of androgen receptor (AR)/MMP13 signals

**DOI:** 10.18632/oncotarget.5638

**Published:** 2015-10-16

**Authors:** ChangYi Lin, WanYing Lin, Shuyuan Yeh, Lei Li, Chawnshang Chang

**Affiliations:** ^1^ George Whipple Lab for Cancer Research, Departments of Pathology and Urology, The Wilmot Cancer Center, University of Rochester Medical Center, Rochester 14642, New York; ^2^ Sex Hormone Research Center, Department of Urology, The First Affiliated Hospital, Xi'an Jiaotong University, Xi'an 710061, China; ^3^ Sex Hormone Research Center, China Medical University/Hospital, Taichung 404, Taiwan

**Keywords:** bladder cancer, neutrophil, microenvironment, androgen receptor

## Abstract

Early studies indicated that several inflammatory immune cells, including macrophages, mast cells, B and T cells in the tumor microenvironment, might influence cancer progression. Here we found that bladder cancer (BCa) cells could recruit more neutrophils than normal bladder cells. The consequences of recruiting more neutrophils might then increase BCa cell invasion *via* up-regulating androgen receptor (AR) signals. Mechanism dissection revealed infiltrating neutrophils could up-regulate AR signals *via* either increased AR mRNA/protein expression or increased AR transactivation. The increased AR signals might then enhance BCa cell invasion *via* increasing MMP13 expression. Together, these results might provide us a new potential therapeutic approach to better battle BCa metastasis *via* targeting the newly identified signaling from infiltrating neutrophils to BCa through AR to MMP13 signals.

## INTRODUCTION

Urothelial carcinoma of the bladder accounts for about 5% of all cancer deaths in humans [1]. Among those cancers, bladder cancer (BCa) is the sixth most common cancer in the United States. Interestingly, it is the third most common cancer in men, but the 11^th^ most common cancer in women [1]. The majority of BCa is non-muscle invasive at diagnosis, and there is a high rate of tumor recurrence and progression even after local surgical therapy [3]. Thus, many patients may require follow-up examinations that include additional prophylactic treatments in the event of recurrence [4]. Therefore, BCa is the most expensive (estimated at #x0024;3.7 billion annually [5]) malignancy to treat in the U.S.

It has become increasingly evident that cancer progression is influenced by the systemic inflammatory response, first described by Virchow in 1876 by demonstrating the presence of leukocytes in neoplastic tissues [6]. Inflammation in the tumor microenvironment plays an important role in the proliferation and survival of malignant cells, as well as promoting angiogenesis and metastasis.

Epidemiological studies indicated that elevated neutrophil-to-lymphocyte ratios are correlated with a poor outcome in various tumors, including BCa [7–11]. Similar to the myeloid macrophages, neutrophils contain a subpopulation of neutrophils named tumor-associated neutrophils (TAN). The potential relationship between TAN infiltration and human cancer prognosis, especially the BCa progression, has not been systematically studied [12].

The androgen receptor (AR) mediates the actions of androgens to affect the development and progression of BCa [13–15]. Using AR knockout (ARKO) mice as an experimental animal BCa model, Miyamoto et al revealed that the AR signals might play a critical role in the development of chemical carcinogen (BBN)-induced bladder carcinogenesis [13, 14]. In our study, we found that BCa could recruit TAN to the BCa that might eventually increase BCa cell invasion *via* modulation of AR/MMP13 signals.

## RESULTS

### BCa cells recruit more neutrophils than non-malignant bladder cells

**Figure 1:**
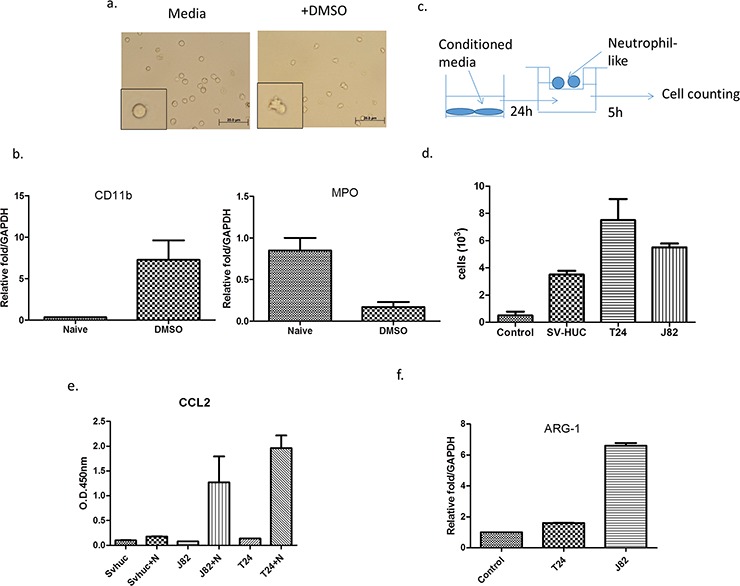
BCa could recruit more N2-like neutrophils than non-malignant bladder cells **a.** We treated HL-60 cells with 1.25% DMSO to become differentiated N2 neutrophils (HL-60N) and **b.** determined the intracellular molecular markers including CD11b and MPO. **c.** A scheme of migration assay. **d.** Conditioned media (CM) from BCa cells could attract more HL-60N cells than CM from non-malignant bladder cells. **e.** *ARG-1* mRNA of neutrophils was upregulated after co-culturing with BCa. **f.** The CM from BCa and HL-60N co-culture contains more CCL2 than CM from non-malignant bladder cell HL-60N co-culture.

We first induced HL-60 into neutrophil-like cells (HL-60N) *via* adding 1.25% DMSO for four days. We then assayed the neutrophil-like phenotype *via* examining the increased CD11b expression and decreased the myeloperoxidase define this (MPO) based on the previous study [17] (Figure 1a–1b).

We then applied the co-culture migration assay [18] using conditioned media (CM) from either muscle-invasive (T24), muscle-invasive (J82) BCa cell lines or a normal bladder cell line (SV-HUC-1), to examine the HL-60N migration. The results revealed that CM from BCa (T24 or J82) could recruit more HL-60N neutrophil-like cells than CM from normal bladder SV-HUC-1 cells (Figure 1c–1d).

Together, results from Figure 1a–1d suggest that BCa cells can recruit more HL-60N cells than normal bladder cells.

We further confirmed that co-cultured neutrophils are N2-type neutrophils *via* examining the chemokine CCL2 expression, as previous studies indicated that the CCL2 secreted by tumors could convert surrounding neutrophils into N2 type neutrophils, named as tumor-associated neutrophils (TAN) [11]. The results revealed that after BCa cells were co-cultured with neutrophils, CCL2 was dramatically increased in the CM, but not after co-culture with normal bladder SV-HUC-1 cells (Figure 1e). Furthermore, the expression of N2 marker Arginase (ARG)-1 [19] was also increased after co-culturing with BCa cells (Figure 1f). These data demonstrate that the neutrophils in the co-culture of BCa-neutrophils are TAN.

### Infiltrated neutrophil cells increase BCa cell invasion

**Figure 2:**
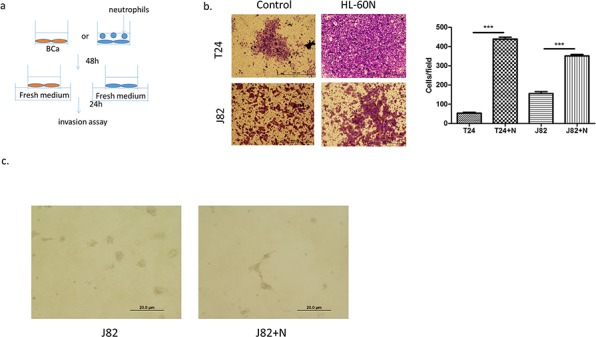
Neutrophils could promote BCa invasion **a.** A scheme of invasion assay. **b.** Microscopic images of invasion assay. (scale bar = 100 μm) and quantitation of the result of invasion assay at right. (*N* = neutrophil; ***p < 0.001) **c.** Microscopic images of 3D invasion assay in J82 (*N* = neutrophil; scale bar = 20 μm).

We next examined the potential effects on BCa progression after recruitment of more neutrophils into BCa cells. We first cultured BCa cells with or without HL-60N cells for 48 h and examined the cell invasion using transwell invasion assays. The results revealed that T24 cells co-cultured with HL-60N cells have 8 fold increased invasive capability (Figure 2a, *p* < 0.01). Similar results were also obtained when we replaced T24 cells with J82 cells or HL-60N cells with PLB-985N cells (Figure 2b *p* < 0.001; Supplmentary Figure S1, S2). Importantly, we also obtained similar results using another 3D invasion assay (Figure 2c). Together, results from Figure 2a–2c suggest that recruited HL-60N cells into BCa cells may increase BCa cell invasion.

### Mechanism dissection how infiltrated neutrophil cells increased BCa cell invasion

**Figure 3:**
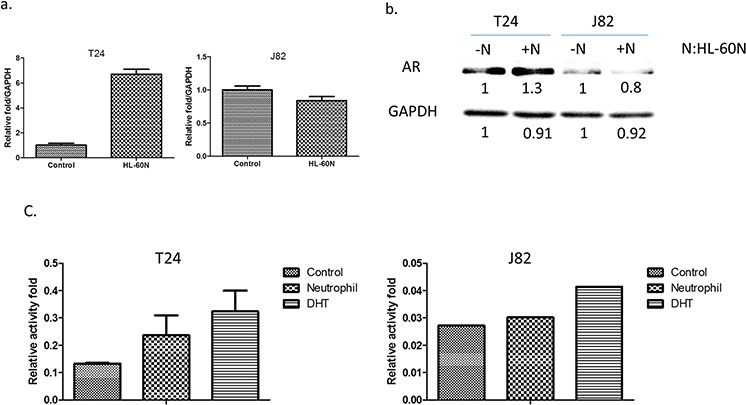
BCa cells express higher level/activity of androgen receptor (AR) after co-culturing with HL-60N **a.** The AR expression at the mRNA level after co-culturing with HL-60N for 48 hours using real-time PCR. **b.** The AR expression at the protein level after co-culturing with HL-60N for 48 hours using western blot. **c.** Androgen response element reporter gene assay.

Early studies indicated that AR might play important roles on BCa [14]. We are interested in examining the potential linkage of AR signals to the infiltrated HL-60N cells-increased BCa cell invasion. As shown in Figure 3a–3b, using real-time PCR and Western blot analysis, we found that co-culturing with HL-60N cells could increase the AR expression at the mRNA and protein levels in BCa T24 cells, (Figure 3a) but not in J82 cells (Figure 3b).

Using ARE-luciferase assay to measure AR transactivation, we found infiltrated neutrophils could increase AR transactivation in T24 and J82 cells (Figure 3c), suggesting infiltrating neutrophils increase AR activity in BCa.

Together, results from Figure 3a–3c suggest that infiltrated neutrophil cells may function through modulating AR signals to increase BCa cell invasion.

### AR is a key factor to mediate infiltrated neutrophils-increased BCa cell invasion

**Figure 4:**
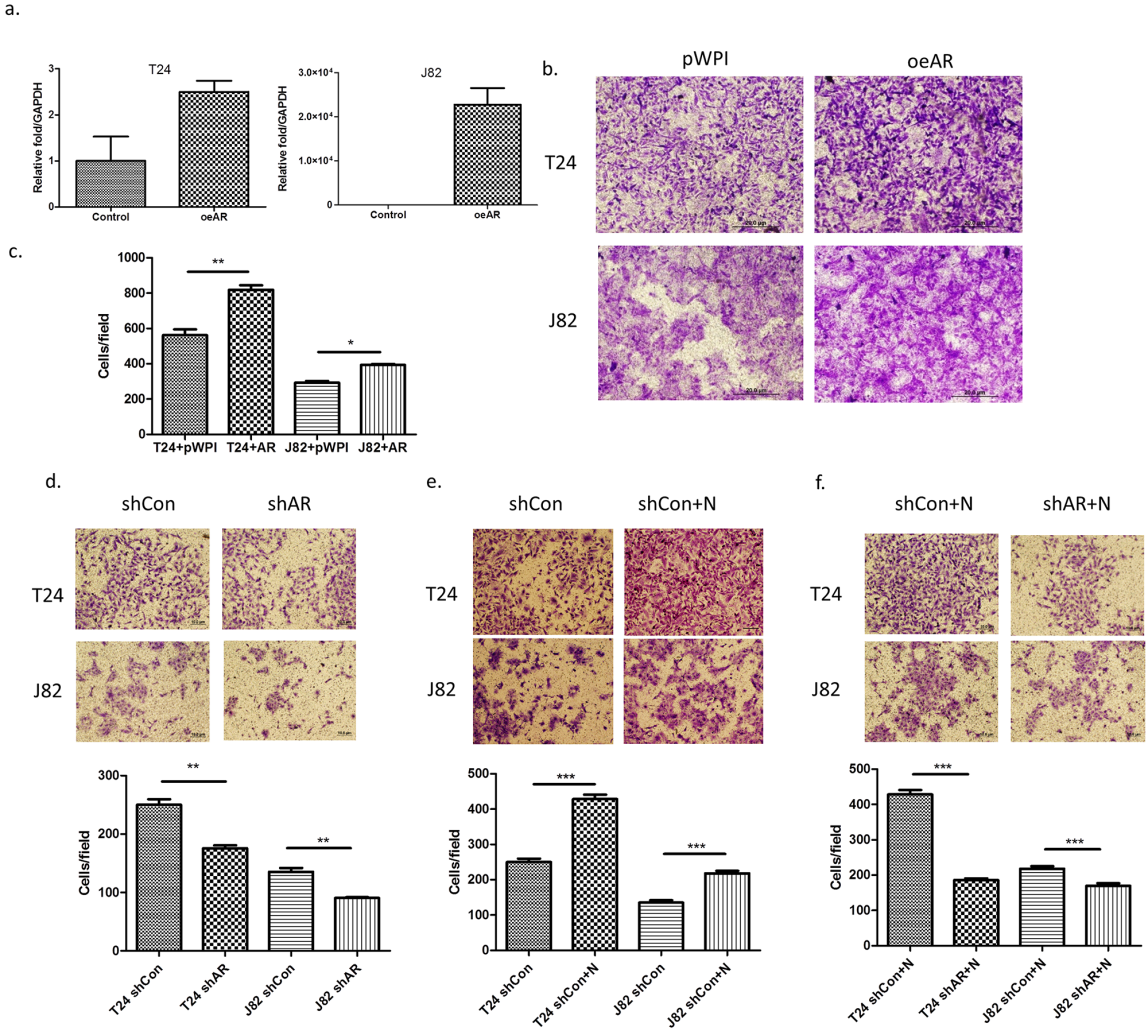
AR is involved in the invasion-promoting effect induced by neutrophils **a.** AR mRNA expression after using lentivirus to overexpress AR (oeAR) in BCa cells. **b.** Microscopic images of invasion assay of cells in a. (scale bar = 20 μm). **c.** Quantitation of the result of invasion assay of Figure 5b. **d.** Microscopic images of BCa invasion assay after knocking down AR (shAR). (scale bar = 20 μm). **e.** Microscopic images of shControl (shCon) BCa invasion assay after co-culturing with neutrophils. **f.** Microscopic images of HL-60N cells co-cultured with BCa cells after BCa after knocking down AR (shAR). Quantitation is below images in d–f. **p < 0,01; ***p < 0.001; pWP1 = vector control; *N* = Neutrophils.

To further demonstrate that infiltrated HL-60N could increase BCa cell invasion *via* altering AR signals, we first added functional AR into T24 and J82 cells and results revealed that higher expressed AR (Figure 4a) led to higher invasive capability in both T24 cells (Figure 4b; *p* < 0.01) and J82 cells (Figure 4c; *p* < 0.05)

We then applied the interruption approach using AR-shRNA lentivirus to knock down AR expression in BCa cells and reduced the BCa cells invasion (Figure 4d; p < 0.01). While infiltrated HL-60N cells increased the BCa cells invasion (Figure 4e; *p* < 0.001), knocking-down AR could then significantly reduce the effect of HL-60N-increased BCa cells invasion (Figure 4f; *p* < 0.001).

Together, results from Figure 4a–4f demonstrated that AR plays an important role to mediate the infiltrated neutrophil-increased BCa cell invasion.

### Mechanism dissection how increased BCa-AR enhanced the infiltrated HL-60N-increased BCa cell invasion

**Figure 5:**
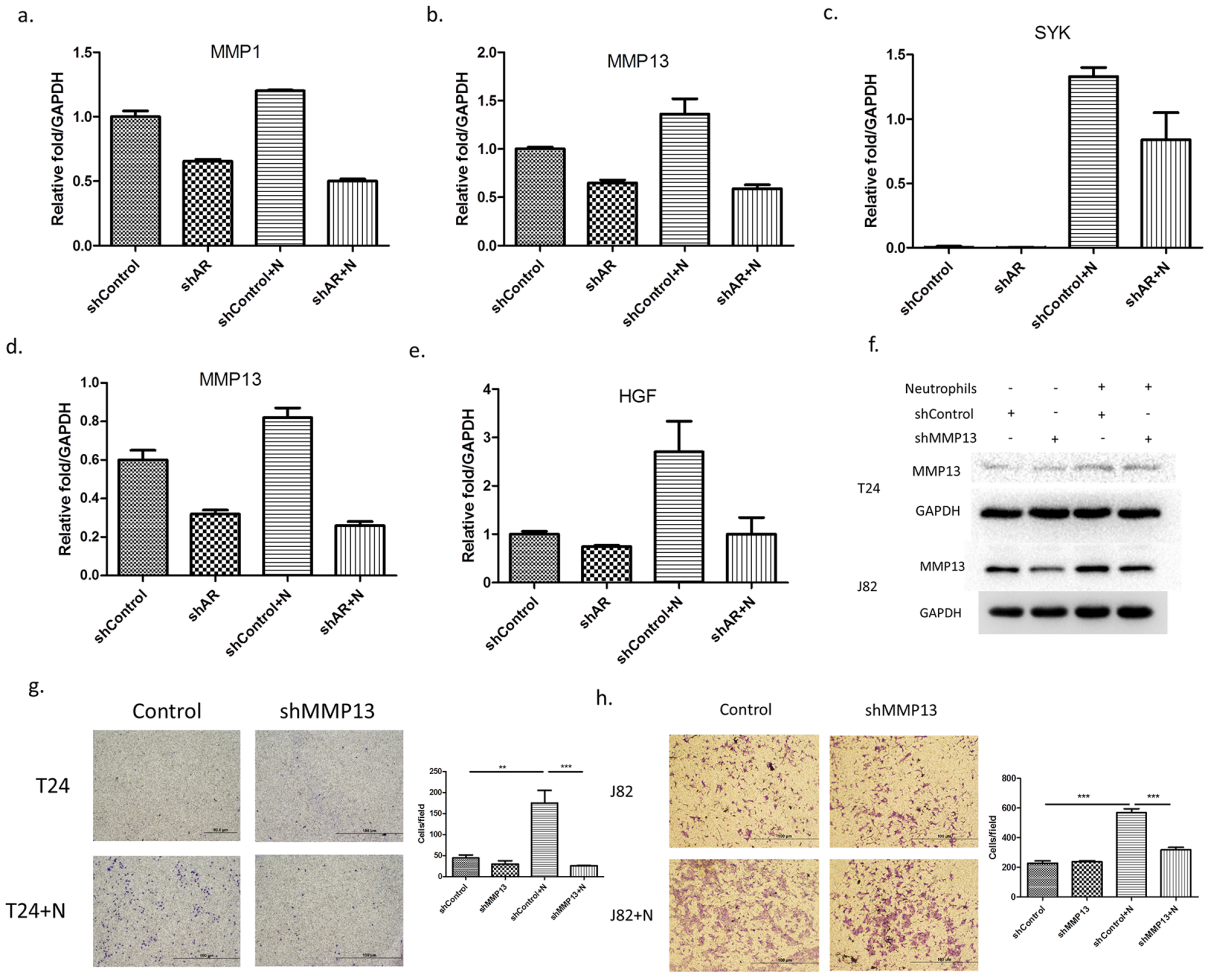
Metastasis-associated genes are involved in the neutrophils-promoting invasion through AR. (a-c.) the expressions The expression of MMP1 (a) MMP13 (b) and SYK (c) were upregulated by co-culturing T-24 cells with HL-60N and inhibited by knocking down AR (shAR). (d-e) The expressions of MMP13 **d.** and HGF **e.** were up-regulated by co-culturing J82 cells with HL-60N and inhibited by knocking down AR in J82. **f.** MMP13 expression level of T24 and J82 cells after co-culture with HL-60N for 48 hours using western blot. (**g-h.**) microscopic images of T24 (g) and J82 (h) cells with and without knocked down MMP13 (shMMP13) and with and without co-culture with neutrophils, quantitations at right. (scale bars = 20 μm) (N: HL-60N). **p < 0,01; ***p < 0.001.

Top further investigate AR associated metastasis-related genes (Supplementary Table S1) in the co-cultured HL-60N and BCa cells, we then added AR-shRNA and found that *MMP1*, *MMP13* and *SYK* genes are up-regulated after co-culture with neutrophils and inhibited by adding AR-shRNA lentivirus in T24 cells (Figure 5a–5c). And *HIF1A* and *MMP13* are up-regulated by adding HL-60N and inhibited by adding AR-shRNA lentivirus in J82 (Figure 5d–5e). We decided to choose MMP13 for further study since its expression was up-regulated in both BCa T24 and J82 cells. Using an interruption approach, we found that infiltrated neutrophils could increase MMP13 expression and adding MMP13-shRNA then reversed the infiltrated neutrophil-induced MMP13 expression (Figure 5f) and infiltrated neutrophil-increased BCa cell invasion in T24 (Figure 5g) and J82 (Figure 5h).

Together, results from Figure 5a–5h demonstrated that the AR/MMP13 axis played an important role to mediate the infiltrated neutrophil-increased BCa cell invasion.

## DISCUSSION

It is of clinical interest to investigate how the BCa tumors interact with infiltrating immune cells from the surrounding the tumor microenvironment (TME), and how the tumor-associated-immune cells increase BCa development. In this study, we found that BCa could recruit more neutrophils to the tumor site than to the normal bladder cells. The consequences of such recruitment indicated more TAN could then increase BCa cell invasion *via* modulation of AR/MMP13 signals.

Early studies indicated that individual cells in the TME might play important roles in tumor progression [20]. For example, fibroblasts might secrete growth factors to increase prostate cancer growth and invasion [21]. Infiltrating macrophages and T cells could increase prostate cancer metastasis through modulation of the CCL2-STAT3 signals or AR-MMP9 signals [22, 23].

Higher neutrophil-to-lymphocyte ratios were also linked to the poor BCa outcome [7, 24], and TAN might function through secreting some cytokines and/or chemokines to influence the TME that resulted in altering the tumor progression. However, both anti-tumor vs pro-tumor paradigms exist for macrophage (M1/M2) [25–27], T-cell (Th1/Th2) polarization [28, 29] and neutrophils (N1/N2) [11]. Suppression of these anti-tumor (N1) neutrophils may impair CD8+ T-cell activation with increased tumor burden [30]. Further studies also suggested that those anti-tumor N1 cells generated in the absence of TGF-β might produce higher levels of TNF-α, MIP-1α, H_2_O_2_ and NO, that were cytotoxic to tumor cells *in vitro* and *in vivo* [11, 30]. In contrast, most TAN appear to have an N2 phenotype and thus contribute to tumor growth and immuno-suppression [30]. Suppression of these pro-tumor N2 neutrophils might then lead to decreased tumor growth [31, 32] and be consistent with our data showing suppression of the infiltrating N2 like HL-60N neutrophils leads to decreased BCa cell invasion. These DMSO-induced differentiated HL-60N neutrophils have been reported to have qualitatively and quantitatively similar responses to chemo-attractants [33] and apocynin [34].

AR has been indicated to play an important role in the development and progression of BCa [14, 35–37]. However, there are controversial results coming from clinical data showing discrepancies in the findings in terms of AR expression in BCa [38–40]. These conflicts may be due to differences in the methods of tissue preparation, experimental conditions, and evaluation system used for immunostaining. In spite of the inconsistent results of AR expression in clinical data, several studies provided promising evidence that androgens promote the proliferation and metastasis of AR-positive BCa cells [14, 36, 37, 41]. Activated AR might promote BCa invasion *via* MMP9 and epithelial-mesenchymal transition [36, 41]. In this present study, our data showed recruited neutrophils could promote invasion *via* modulating AR/MMP13 signaling pathway by silencing AR or MMP13.

MMP13 was first identified in breast carcinoma [42]. Compared with the other MMPs, MMP13 has wide substrate specificity and a limited expression pattern [43]. Expression of MMP13 is observed to be limited to tissues undergoing rapid connective tissue remodeling, such as during fetal bone development, post-natal bone remodeling and gingival wound repair [44]. However, MMP13 is also expressed in various diseases involving degradation of collagenous exrtracellular matrix and in malignant tumors, such as squamous cell carcinomas of the head and neck, cutaneous basal-cell carcinomas, chondrosarcomas and melanomas [45]. In BCa, it was demonstrated that MMP13 was expressed little in normal bladder cells yet it was expressed in tumor cells, particularly at the invading edges [46], suggesting that MMP13 may serve as a marker for tumor transformation and invasion and can be a potential therapeutic target. Our results showing that recruited neutrophils increased BCa cell invasion *via* up-regulation of AR/MMP13 signals are in agreement with early findings.

In summary, finding a new signal from infiltrating neutrophils to BCa through AR to MMP13 to increase BCa cell invasion not only strengthens the importance of AR roles in BCa progression, it may also provide a potential therapeutic approach to better suppress BCa metastasis.

## MATERIALS AND METHODS

### Cell culture and stable cell lines

The human BCa cell lines T24 (with high endogenous AR) and J82 (with low endogenous AR) as well as normal human uroepithelium cell line, SV-HUC-1 were purchased from ATCC and cells were maintained in Dulbecco's Minimum Essential Medium (DMEM) (Invitrogen, Carlsbad, CA) with 10% fetal bovine serum (FBS) and 1% antibiotics. The immature neutrophils, HL-60 and PLB-985, were stimulated with 1.25% DMSO for 1 weeks to be differentiated into N2 type neutrophils, named HL-60N and PLBN, and were maintained in 10% heat-inactivated FBS containing RPMI media with antibiotics. All cell lines were cultured in a 5% (v/v) CO_2_ humidified incubator at 37°C. The AR knockdown and overexpressing BCa cells wee established by lentiviral transduction of siAR or AR cDNA, respectively.

### Lentiviral expression plasmid construction and virus production

ShAR (PLKO.1-puro-shAR) was constructed with target sequence 5′-GTCGCGACTACTACAACTT-3′ and shMMP13 was constructed with target sequence 5′-CCAACCGTATTGATGCTGC-3′ according to Addgene's pLKO.1 protocol. Lentiviral particles were generated by calcium phosphate transfection of lentiviral expressing, packaging and envelop plasmids into HEK293T cells. Lentiviral particles were collected to infect target cells according to a previous report [16].

### Invasion and migration assay

For *in vitro* invasion assays, the upper chambers of the transwells (8 μm pore size) were pre-coated with the growth factor-reduced matrigel (matrigel: serum free DMEM = 1:15) (BD Biosciences). Before invasion assays, 1 × 10^6^ BCa cells were co-cultured with 1 × 10^5^ HL-60N cells for 2 days in 10-cm dishes. The parental BCa cells or co-cultured BCa (1 × 10^5^) were plated onto the upper chambers of the transwells. After 24 hrs incubation, the cells in the upper chambers were removed. The insert membranes were fixed with methanol, stained with crystal violet, and the positively stained cells invaded to the under part of the membranes were counted. The invaded BCa cell numbers were averaged from counting numbers of five random fields. Each data point was run in triplicate and each set of experiments were performed in triplicate for calculation as mean ± SEM. For 3D invasion, we thawed Matrigel on ice and added 40 μl to each well of 8-well glass chamber slide (at 50 μl/cm2) and spread the Matrigel evenly. Place the slides in the cell culture incubator and allow the Matrigel to solidify (takes 15–20 min). Plate 1 × 10^4^ J82 cells after co-culture with HL-60N into each well with media containing 5% Matrigel and 10 ng/ml EGF. We changed media every 3 days with assay media containing 2.5% Matrigel and 5.0 ng/ml EGF. J82 cells take about 7 days to form protrusion structures. 10 different fields under 200x microscope were chosen randomly to count the number of protrusions on cells in each field. For *in vitro* migration assay, the 1 × 10 [5] HL-60N were plated onto the 6 μm pore size upper chambers of transwells. The CM of various co-cultured cells was placed into bottom well to attract HL-60N. After 5 hrs incubation, the HL-60N were collected and calculated from the bottom well.

### Quantitative PCR

Total RNA was extracted from each cell line using Trizol (Invitrogen). Reverse transcription was performed using the iScript reverse transcription kit (Bio-Rad). Quantitative real-time PCR (qRT-PCR) was conducted using a Bio-Rad CFX96 system with SYBR green to determine the levels of mRNA expression of listed genes. Expression levels were normalized to the expression of GAPDH mRNA.

### Western blot assay

Cells were washed twice in PBS and lysed with RIPA buffer containing 1% protease inhibitors (Amresco, Cochran, USA). Protein concentrations in the cell lystate solutions were determined by BCA protein assay (Amresco, Cochran, USA). Each cell lystate was mixed with 5 × SDS-PAGE loading buffer (Amresco). Equivalent protein quantities were loaded to 10% SDS-polyacrylamide gels (Bio-Rad). Proteins were electotransferred to PVDF membranes (Millipore, Atlanta GA, USA) that were blocked in Tris-buffered saline plus 0.05% Tween-20 (TBS-T) containing 5% non-fat dried milk for 1 hr. The membranes were washed in TBS-T and incubated with each primary monoclonal antibody overnight at 4°C. The following primary antibodies were used: rabbit anti-AR polyclonal antibody, and mouse anti-GAPDH monoclonal antibody (Santa Cruz) and mouse anti-MMP13 monoclonal antibody were used at 1:1,000 dilution. The immuno-positive bands were visualized with an ECL chemiluminescent detection system (Thermo Scientific), and the images were transferred to the Bio-Rad imaging system. All analyses were performed at least in duplicate.

### Statistical analysis

Data are presented as mean ± SEM from at least 3 independent experiments. Statistical analyses involved paired *t*-test with SPSS 17.0 (SPSS Inc., Chicago, IL). *In vivo* study measurements of tumor metastasis among the three groups were analyzed through one-way ANOVA coupled with the Newman-Keuls test. *P* < 0.05 was considered statistically significant.

## SUPPLEMENTARY FIGURES AND TABLE


