# Supplementary material for: Infiltrating neutrophils increase bladder cancer cell invasion via modulation of androgen receptor (AR)/MMP13 signals
Source: Oncotarget. 2015 Oct 16;6(40):43081–9. doi: 10.18632/oncotarget.5638 (PMC4767492; doi:10.18632/oncotarget.5638)
Supplement: Supplementary file 1 [file oncotarget-06-43081-s001.pdf]

SUPPLEMENTARY FIGURES AND TABLE

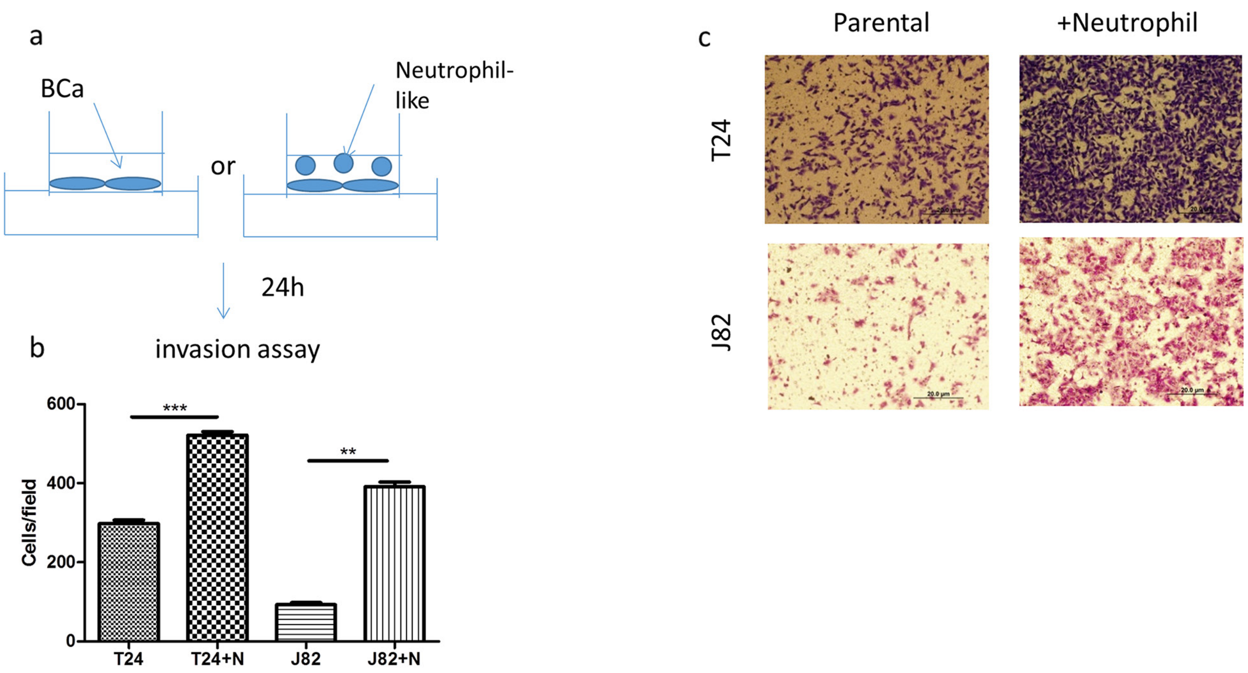

Supplementary Figure S1: Neutrophils could promote BCa invasion. a. A scheme of invasion assay. **b.** Quantitation of the results of invasion assay in c. **c.** Microscopic images of invasion assay. (scale bar = 20 μm). (N = neutrophil ***p* < 0.01; ****p* < 0.001).

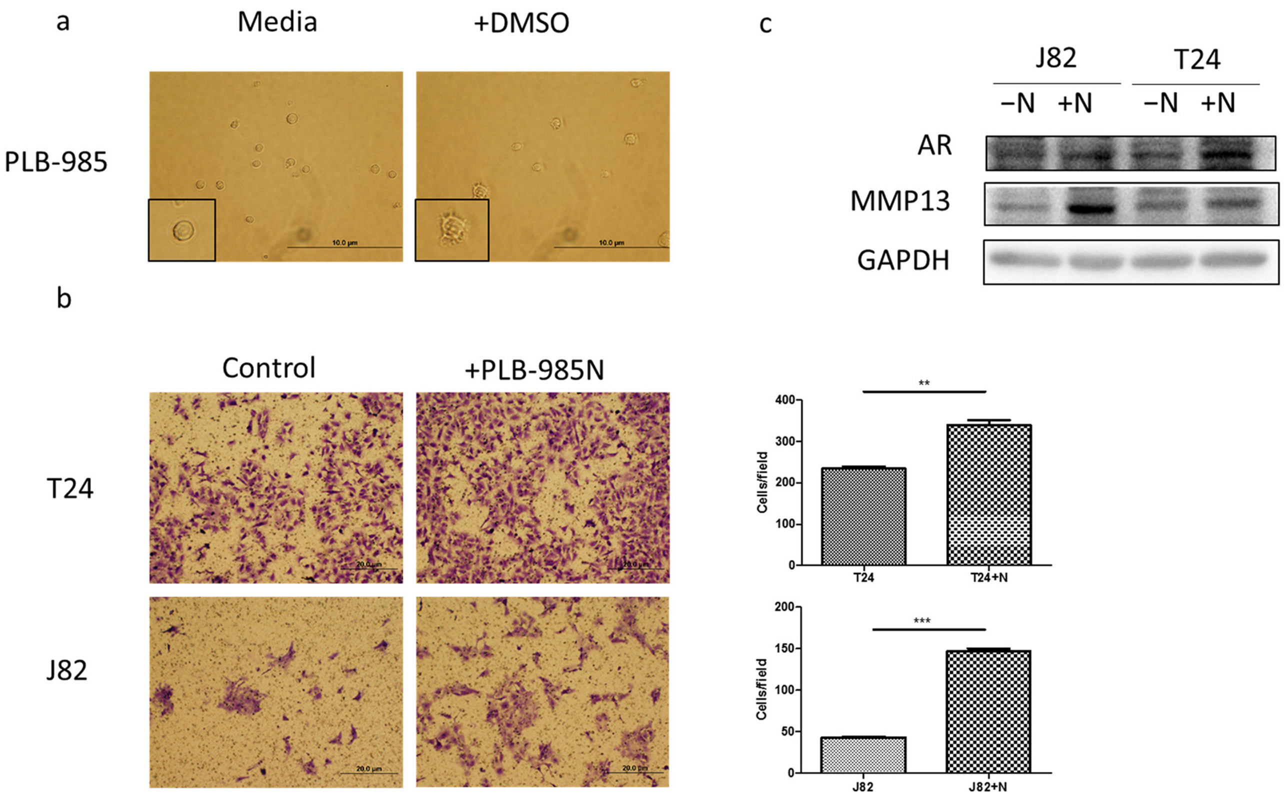

Supplementary Figure S2: PLB-985-derived neutrophils (PLB-985N) could promote BCa invasion. a. Morphology change after treating cells with 1.25% DMSO (scale bar = 10 µm); **b.** Microscopic images of invasion assay (scale bar = 20 µm) and quantitation at right of the results of invasion assay. (**$p < 0.01$; ***$p < 0.001$). **c.** BCa cells express higher level of AR/MMP13 after co-culturing with PLB-985-derived neutrophils. (N = PLB-985N).

Supplementary Table S1: Metastasis-related genes screening

Unigene	Symbol	T24	J82
		Change fold (SEM)	Change fold (SEM)
Hs.508716	COL4A2	1.5(0.1)	1(0.1)
Hs.124503	ZEB1	2.1(0.1)	1.2(0.0)
Hs.396530	HGF	3(0.5)	1.3(0.3)
Hs.44227	HPSE	1.8(1.3)	1(0.0)
Hs.160562	IGF1	0.9(0.2)	5.8(0.5)
Hs.83169	MMP1	2.8(0.2)	0.9(0.2)
Hs.83169	MMP13	1.4(0.2)	1.4(0.4)
Hs.513617	MMP2	1.1(0.1)	0.7(0.1)
Hs.297413	MMP9	0.5(0.3)	0.9(0.1)
Hs.2399	MMP14	3.3(0.1)	1.1(0.0)
Hs.727986	SMAD3	2.1(0.1)	0.9(0.0)
Hs.645227	TGFB1	3.3(0.3)	1(0.1)
Hs.522632	TIMP1	1.3(0.1)	0.9(0.0)
Hs.633514	TIMP2	3(0.2)	1.1(0.1)
Hs.73793	VEGF	3.2(0.2)	1.1(0.1)
Hs.476018	CTNNB1	1.5(0.0)	0.9(0.1)
Hs.502328	CD44	1.1(0.0)	1.3(0.1)
Hs.522378	DAB2IP	2.8(0.2)	0.9(0.1)
Hs.444082	EZH2	0.6(0.0)	1.1(0.1)
Hs.37003	HRAS	1.1(0.0)	0.9(0.0)
Hs.95008	KISS-1	0.4(0.0)	0.8(0.2)
Hs.132966	MET	1.5(0.0)	1(0.1)
Hs.202453	MYC	1.1(0.0)	1.1(0.1)
Hs.9235	NME4	1(0.0)	1.1(0.0)
Hs.195659	SRC	2.7(0.0)	1.1(0.1)
Hs.371720	SYK	3.7(0.4)	1(0.1)
Hs.512592	TP53	2.1(0.1)	1(0.0)
Hs.124503	ZEB1	1.5(0.0)	1.1(0.2)
Hs.418123	CTSL1	1.2(0.0)	0.9(0.0)
Hs.597216	HIF1A	1.1(0.1)	1(0.0)
Hs.468410	HIF2A	2.7(0.1	0.9(0.0)
Hs.527778	KAI1	1.9(0.1)	1(0.2)
Hs.444986	METAP2	1.1(0.0)	1.1(0.0)
Hs.525629	MTA1	1.7(0.1)	0.8(0.0)
Hs.37982	NEDD9	1(0.1)	0.9(0.0)
Hs.55279	SERPINB5	0.9(0.0)	0.9(0.0)

(*Continued*)

Unigene	Symbol	T24	J82
		Change fold (SEM)	Change fold (SEM)
Hs.351316	TM4SF1	0.7(0.0)	0.8(0.0)
Hs.435215	FLT4	ND	ND
Hs.155983	JMJDA	ND	ND

The expression change fold of metastasis-related genes in T24 and J82 after coculturing with HL-60N. (change fold = co-culture/control).
ND: not detectable.
SEM: standard error of the mean.
